# Genomes of Two New Ammonia-Oxidizing Archaea Enriched from Deep Marine Sediments

**DOI:** 10.1371/journal.pone.0096449

**Published:** 2014-05-05

**Authors:** Soo-Je Park, Rohit Ghai, Ana-Belén Martín-Cuadrado, Francisco Rodríguez-Valera, Won-Hyong Chung, KaeKyoung Kwon, Jung-Hyun Lee, Eugene L. Madsen, Sung-Keun Rhee

**Affiliations:** 1 Department of Microbiology, Chungbuk National University, Cheongju, South Korea; 2 Department of Biology, Jeju National University, Jeju, South Korea; 3 Departmento de Producción Vegetal y Microbiología, Evolutionary Genomics Group, Universidad Miguel Hernández, Alicante, Spain; 4 Korean Bioinformation Center, KRIBB, Yuseong-gu, Daejeon, South Korea; 5 Korea Institute of Ocean Science and Technology, Ansan, South Korea; 6 Department of Microbiology, Cornell University, Ithaca, New York, United States of America; Université Claude Bernard - Lyon 1, France

## Abstract

Ammonia-oxidizing archaea (AOA) are ubiquitous and abundant and contribute significantly to the carbon and nitrogen cycles in the ocean. In this study, we assembled AOA draft genomes from two deep marine sediments from Donghae, South Korea, and Svalbard, Arctic region, by sequencing the enriched metagenomes. Three major microorganism clusters belonging to *Thaumarchaeota*, *Epsilonproteobacteria*, and *Gammaproteobacteria* were deduced from their 16S rRNA genes, GC contents, and oligonucleotide frequencies. Three archaeal genomes were identified, two of which were distinct and were designated *Ca*. “Nitrosopumilus koreensis” AR1 and “Nitrosopumilus sediminis” AR2. AR1 and AR2 exhibited average nucleotide identities of 85.2% and 79.5% to *N*. *maritimus*, respectively. The AR1 and AR2 genomes contained genes pertaining to energy metabolism and carbon fixation as conserved in other AOA, but, conversely, had fewer heme-containing proteins and more copper-containing proteins than other AOA. Most of the distinctive AR1 and AR2 genes were located in genomic islands (GIs) that were not present in other AOA genomes or in a reference water-column metagenome from the Sargasso Sea. A putative gene cluster involved in urea utilization was found in the AR2 genome, but not the AR1 genome, suggesting niche specialization in marine AOA. Co-cultured bacterial genome analysis suggested that bacterial sulfur and nitrogen metabolism could be involved in interactions with AOA. Our results provide fundamental information concerning the metabolic potential of deep marine sedimentary AOA.

## Introduction

Aerobic nitrification is a key process in the nitrogen cycle that converts ammonia to nitrate via nitrite and is catalyzed by aerobic autotrophic ammonia-oxidizing and nitrite-oxidizing microorganisms. The first step in autotrophic nitrification, the oxidation of ammonia, was long thought to be exclusive to *Proteobacteria* in the domain *Bacteria* [1]; however, more recently, metagenomic analyses of terrestrial [2] and marine environments [3] revealed that ammonia oxidation is also associated with *Archaea*. Moreover, critical evidence for the existence of autotrophic ammonia-oxidizing archaea (AOA) was obtained through characterization of the first ammonia-oxidizing archaeon, *Nitrosopumilus maritimus* SCM1, which was isolated from a marine aquarium [4]. This discovery was followed by the successful cultivation of diverse AOA of *Thaumarchaeota* [5], [6] from marine (group I.1a) [4], [7], [8] and soil (group I.1a and I.1b) [9]–[11] environments. Furthermore, molecular ecological studies indicate that AOA often predominate over ammonia-oxidizing bacteria in marine environments such as the North Sea and coastal sediments [8], [12].

The seafloor comprises approximately two-thirds of the Earth’s surface and is therefore one of the most extensive of all microbial habitats. Quantitative assessments of subsurface microbial populations indicate that prokaryotes constitute a large portion of the Earth’s overall biomass, and that marine sediment processes may therefore substantially contribute to the global nitrogen budget. Research into nitrification, a key step in the nitrogen cycle, has focused on water-column, and studies regarding marine sediment nitrification are minimal. Investigations into the metabolic properties and nitrification potential of sedimentary AOA are therefore necessary to understand the nitrogen cycle in marine environments.

Fundamental information about microorganisms and their metabolic features can be revealed via metagenomic and genomic techniques. Analysis of the genome sequence of an *amoA*-encoding archaeon *Ca*. “Cenarchaum symbiosum” from a marine sponge [13], [14] and a marine ammonia-oxidizing archaeon *N*. *maritimus* [15] provided valuable insights into the evolution of nitrogen and carbon metabolism in marine AOA of the *Nitrosopumilus* lineage (also called group I.1a). Comparative analyses of group I.1a AOA genome sequences from low-salinity aquifers and terrestrial environments have revealed several genetic traits likely to be adaptations to such habitats, such as motility and protection from osmotic stress [16], [17]. AOA metagenomic information obtained from the water column of the Gulf of Maine has shed light on the metabolic potential of planktonic AOA [18]. Although the genomes of two AOA enriched from low-salinity sediments have been sequenced [19], [20], genomic data from deep marine sedimentary AOA are not yet available.

AOA are widespread and dominant ammonia-oxidizers in marine sediment [12]. One of the main difficulties in obtaining axenic AOA cultures is their dependence on co-cultured bacteria, as described in AOA characterization reports [10], [11], [21], [22]. Sedimentary AOA were, however, successfully enriched when co-cultured with sulfur-oxidizing bacteria (SOB) in a technique that facilitated characterization of the AOA [7]. Here, we analyzed metagenomes from enrichment cultures and were able to assemble the genomes of two deep marine sedimentary AOA. The aims of this study were to investigate the genomic features of deep marine sedimentary AOA through comparisons with the genomes of other AOA and to assess possible microbial interactions between deep marine sedimentary AOA and co-cultured bacteria.

## Results and Discussion

### Metagenome analysis, assembly, and binning

We obtained 536.8 Mb and 308.2 Mb of metagenomic sequences from two independently enriched ammonia-oxidizing cultures containing thaumarchaeotal group I.1a archaeal strains, named AR (from Svalbard, Arctic region) and SJ (from Donghae, South Korea), respectively. General features of the metagenome datasets are as indicated in Table S1. The GC% profiles of the raw reads from the two enrichment metagenomes were very similar to one another (Figure S1).

Single reads of 16S rRNA genes recovered from the metagenome dataset (n = 1,100 in AR and n = 908 in SJ cultures) were used to analyze the compositions of the microbial communities that were enriched in the two cultures (Figure S2). The most frequently recovered 16S rRNA gene sequences were affiliated to *Epsilonproteobacteria* (60–62%), *Thaumarchaeota* (13–17%), and *Gammaproteobacteria* (10–18%), with the proportions of these three taxa being similar in the two cultures (Figure S2). Most of the 16S rRNA gene sequences of *Epsilonproteobacteria* were affiliated with the sulfur-oxidizing genus *Sulfurovum*. More than 10% of the 16S rRNA gene reads from each metagenome were affiliated with *Thaumarchaeota*, and, specifically, the genus *Nitrosopumilus*. *Gammaproteobacteria* sequences were related to those of diverse *Gammaproteobacteria* (e.g., *Marinobacter*, *Marinobacterium*, and *Neptuniibacter*). Overall, this analysis suggested that the proportion of 16S rRNA genes from archaea was approximately 20%, which was lower than the proportion of archaea observed by previous fluorescence *in situ* hybridization analysis of the SJ and AR cultures [7]. This discrepancy could have arisen due to the presence of multiple rRNA operons in bacterial genomes [23] by contrast with the single rRNA operon in the genome of *N*. *maritimus* (*Thaumarchaeota*) [15]. Indeed, Nakagawa et al. [24] reported that the genome of *Sulfurovum* sp. NBC37-1 (*Epsilonproteobacteria*), a close relative of the dominant bacterium in the SJ and AR cultures, has three copies of the rRNA operon. Data obtained from 16S rRNA gene reads were complemented by comparing the entire metagenome dataset of functional genes to homologous genes of known microbial genomes using the MG-RAST server (Figure S3).

**Figure 1:**
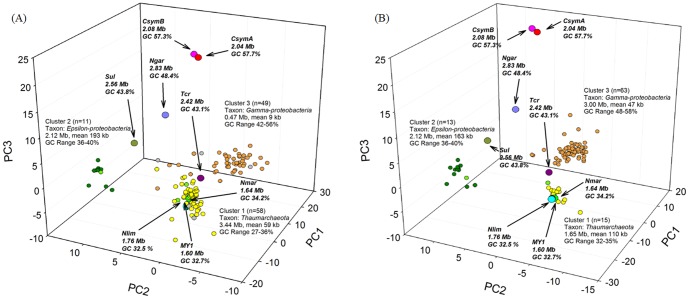
Principal component analysis of oligonucleotide frequencies in assembled contigs from two archaeal enrichment cultures (A) AR culture, and (B) SJ culture. Reference genomes are shown as larger circles. The total number of contigs for each group (*Gammaproteobacteria*, *Epsilonproteobacteria,* and *Thaumarchaeota*), total length, mean length, and GC content range are also indicated. The contig types and published genomes are as follows: orange, *Gammaproteobacteria*; yellow, *Thaumarchaeota*; green, *Epsilonproteobacteria*; light green, assembled contigs including viral coding sequences; gray, not identified; red, *Ca*. “Cenarchaum symbiosum” A (CsymA); fuchsia, *Ca*. “C. symbiosum” B (CsymB); lime, *Nitrosopumilus maritimus* SCM1 (Nmar); blue, *Ca*. “Nitrosoarchaeum koreensis” MY1 (MY1); cyan, *Ca*. “Nitrosoarchaeum limnia” (Nlim); violet, *Ca*. “Nitrososphaera gargensis” (Ngar); teal, *Sulfurovum* sp. NBC37-1 (Sul); and purple, *Thiomicrospira crunogena* XCL-2 (Tcr).

Assembly of the metagenomic data produced 15,155 and 2,595 contigs from the AR and SJ metagenomic sequences, respectively (Table S1). We filtered the contigs, selecting only those that were ≥ 5 Kb in length (n = 118 for AR and n = 91 for SJ) and which yielded consistent hits to a single high-level taxon (e.g., *Thaumarchaeota*, *Epsilonproteobacteria* and *Gammaproteobacteria*). An examination of GC% versus length in the selected contigs indicated they comprised three clusters (Figure S4). Moreover, principal component analysis of the oligonucleotide frequencies also revealed three distinct clusters in each enriched sample (Figure 1). Based on BLAST analysis of the genes, we assigned clusters 1, 2, and 3 to *Thaumarchaeota*, *Epsilonproteobacteria*, and *Gammaproteobacteria*, respectively, which was consistent with results obtained from the 16S rRNA analysis (Figure S2). The GC% range in cluster 1 (*Thaumarchaeota*) (Figure 1) was similar in both the AR and SJ assemblies (27–37% in AR and 32–35% in SJ). With the exception of *Ca*. “C. symbiosum” (57%) [13] and *Ca*. “Nitrososphaera gargensis” (48%) [25], all other previously analyzed AOA, including *N*. *maritimus*, had GC contents of 32–34% [15]–[17]. The amounts of sequence obtained for cluster 1 differed between the two clusters: 3.44 Mb in AR and 1.65 Mb in SJ. Considering the size of the *N*. *maritimus* genome (1.64 Mb), the 1.65 Mb size of the archaeal cluster from the SJ metagenome assembly potentially represented a draft genome of a single AOA. However, the 3.44 Mb of contigs in cluster 1 of the AR metagenome suggested that two putative archaeal draft genomes had been assembled.

The GC content of cluster 2 was approximately 43%, which corresponded to that of *Sulfurovum* sp. NBC37-1 (43.8%) [24]. The expected genome size of cluster 2 (2.12 Mb) was slightly smaller than that of *Sulfurovum* sp. NBC37-1 (2.56 Mb). We were unable to detect the 16S rRNA gene within cluster 3, which contained the gammaproteobacterial contigs, and so were unable to definitively determine phylogenetic position. BLAST analysis indicated that cluster 3 contig genes were most similar to genes in *Gammaproteobacteria* genomes such as *Oceanospirillum*. The Average Nucleotide Identity (ANI) [26] of the gamma- and epsilonproteobacterial clusters in the two metagenome sets indicated that they were nearly identical (∼99%). Some features of the binned contigs from both metagenomic datasets are summarized in Table 1.

**Table 1:** Features of binned contigs for genomes of thaumarchaeota, epsilon- and gammaproteobacteria (≥ 5 Kb contigs).

	*Thaumarchaeota*		*Epsilonproteobacteria*		*Gammaproteobacteria*	
	AR	SJ	AR	SJ	AR	SJ
Size (Mbp)	3.44	1.65	2.12	2.12	0.47	3.00
No. of predicted genes	4,148	1,934	2,136	2,138	512	2,907
No. of contigs	58	15	11	13	49	63
Average contig size (Kb)	59	110	193	163	9	47
Average GC content (%)	33.83	34.31	39.37	39.39	52.37	53.42
Average gene length (bp)	737	760	903	903	818	924
Coding percentage (%)	88.9	89.1	91.0	91.0	89.4	89.7
Genome coverage (X)	34	42	71	67	7	12
RNA genes						
23S	2	1	ND	ND	ND	ND
16S	2	1	ND	ND	ND	ND
5S	2	1	ND	ND	ND	ND

ND, not detected.

### Establishing draft genome assemblies for three deep marine sedimentary archaea and defining their unique characteristics

The binning and assembly procedures described above were used to define three AOA draft genomes. We hypothesized that the cluster 1 (thaumarchaeotal) sequences from culture AR (3.44 Mb) represented two genomes, henceforth termed AR1 and AR2. Cluster 1 sequences from culture SJ (1.65 Mb) appeared to represent a single genome.

Genomic diversity in a microbial population can be determined by analyzing sequence variations in metagenome reads. We used the Strainer program (http://www.bioinformatics.org/strainer/wiki/) to assess variation in the archaeal populations of the metagenome datasets. Archaeal diversity in the AR and SJ cultures was assessed by analyzing the ammonia monooxygenase gene (ammonia monooxygenase alpha subunit, *amoA*), which is involved in ammonia oxidation, and the 16S-23S rRNA intergenic spacer (ITS) region. The *amoA* and ITS sequences were examined in raw reads (data not shown), and the results fully supported the above hypothesis that the metagenomic data captured a single draft archaeal genome in the SJ culture and two draft archaeal genomes in the AR culture. Archaeal contigs in the AR culture clearly separated into two distinct groups based on contig alignment with *N*. *maritimus* using Mauve [27] and ANI analysis with *N*. *maritimus*.

We propose that our assembled genomes warrant draft genome status for the following reasons: (i) Each draft genome features 97–98% of the archaeal genes used by the NIH Human Microbiome Project as criteria for complete draft genomes (http://hmpdacc.org/tools_protocols/tools_protocols.php) [28]. These archaeal genes are known to be highly conserved between the genomes of free-living *Archaea* and comprise 104 core gene groups. Additionally, the majority of the core archaeal genes are found in the complete or nearly complete genomes of several published AOA (*Ca*. “C. symbiosum”, 92%; *Ca*. “Na. koreensis”, 98%; *N*. *maritimus*, 100%; and one exception, *Ca*. “N. gargensis”,74%); (ii) The two draft genomes of SJ and AR1 were independently sequenced and assembled but were nearly identical to one other, as recognized by gene content and synteny comparisons; (iii) A high degree of genomic similarity was observed between the three draft archaeal genomes and the completed *N*. *maritimus* genome. Furthermore, the number of tRNAs (n = 44) was identical in the draft genomes of SJ, AR1, and AR2, and the complete genome of *N*. *maritimus*.

The two AR1 and AR2 archaeal genomes exhibited approximately 80% ANI with each other and ANIs of 85.2% and 79.5% with *N*. *maritimus*, respectively. The ANI of the AR1 archaeal bins with those of the SJ culture was ∼99%; no significant differences were observed between the SJ and AR1 archaeal contigs with respect to gene content or local synteny. On the basis of these results, we concluded that the SJ and AR1 assembled archaeal genomes were indistinguishable and might have originated from very closely related microorganisms. Therefore, our further analyses focused on two of the three archaeal genomes: AR1 (synonymous with the archaeon from culture SJ) and AR2.

Despite the strong similarities (>99.5%) between the 16S rRNA gene sequences in *N*. *maritimus* and in the AOA obtained from our enrichments (Table S2 and Figure S5), the low ANI (<85%) indicates high genomic variation within this cluster of marine AOA. The proposed cutoff for defining separate species is 94% ANI between two genome sequences [26]. This criterion suggests that each archaeal strain (AR1 and AR2) can be considered a separate species distinct from *N*. *maritimus*. We propose that these genomes represent two new marine AOA within the genus *Nitrosopumilus,* named *Ca*. “Nitrosopumilus koreensis” (AR1 and SJ) [29] and “Nitrosopumilus sediminis” (AR2) [30].

### Genetic differences between AOA genomes and their adaptive implications

Most of the putative coding sequences (CDS) in the AR1 and AR2 genomes (71.9% and 65.1%, respectively) had homology to *N*. *maritimus* genes, and most of the genes were syntenic with those in the *N*. *maritimus* genome (Figure S6). However, 20.5% and 24.4% of the putative CDS of the AR1 and AR2 genomes, respectively, had no similarity to genes in other known organisms.

We hypothesized that the adaptive traits of deep sedimentary AOA in our enrichment cultures might contrast with those of water-column AOA. To address this, a recruitment analysis was performed in which nucleotide-sequence fragments from the planktonic Sargasso Sea metagenome dataset of the global ocean sampling (GOS) database [3] were mapped onto the AR genomes (Figure S7). Many of the genes that were present in the AR genomes but absent in the Sargasso Sea metagenome dataset were clustered in genomic islands (GIs) of >15 Kb (Figure S7, and Tables S3 and S5).

GIs were a major feature of the AR1 and AR2 genomes (Tables S3 and S5) and comprised approximately 15% of the total AR1 (six GIs) and AR2 genomes (12 GIs). Most of the GIs in the AR1 and AR2 genomes were different from one another and were absent from the *N*. *maritimus* genome, and gene functions can be putatively inferred for approximately half of the genes in the GIs. Most GI genes in both the AR1 and AR2 genomes were related to cell-wall biosynthesis, osmotic stress tolerance, antibiotic resistance, sensory signal transduction, and phage proteins. In addition, the GIs of both genomes comprised genes with high anomalies in codon usage, indicating that they might have been obtained via horizontal transfer events, as suggested by Rusch et al. [31].

The Clusters of Orthologous Genes (COG) classification of the GI genes from the two genomes indicated that genes belonging to COG class M (cell wall/membrane/envelope biogenesis), K (transcription), and T (signal transduction mechanisms) were abundant (Figure S8). This is in partial contrast to the COG classes found in the GIs of other archaeal genomes, which are predominantly M or Q (secondary metabolite biosynthesis, transport, and catabolism) [32]. The proteinaceous surface layers of AOA have an abundance of reactive surface sites that are conceivably related to their oligotrophic adaptations [33]. The frequent observation of COG class M genes in the GIs of the AR1 and AR2 genomes could contribute to variations in cell surface structure, which might be important factors for niche specialization in AOA ecotypes. Overall, the identified GIs might constitute strain-specific (hyper)variable regions or sedimentary AOA-specific regions.

### Ammonia oxidation, electron transfer, and carbon fixation for the deep marine sedimentary AOA

Pathways for ammonia oxidation, electron transport, and carbon fixation were assembled from the AR1 and AR2 archaeal genomes and compared with other reference AOA genomes. The AR1 and AR2 archaeal strains held key metabolic traits in common with other AOA, including *N*. *maritimus* (Table S4).

#### Ammonia oxidation and electron transport chain

All of the putative ammonia monooxygenase genes (*amo*; *amoA*, *amoB*, and *amoC*) were found in the AR1 and AR2 genomes. The gene arrangement [*amoA*-hypothetical gene (named *amoX*)-*amoC*-*amoB*] was similar to that in other AOA of the *Nitrosopumilus* cluster (e.g., *N*. *maritimus*) as well as into *Ca*. “N. devanaterra” [34], but differs from the gene arrangements in group I.1b AOA [9],[25]. For example, the *amo* genes in some group I.1a marine lineages and in most of the soil lineages (group I.1b) were not consecutive, but were interrupted by other genes. In most AOA, another small protein encoding a transmembrane protein and referred to as *amoX* was linked to the *amoA* gene [35].

Although AOA produce nitrite as the final product of ammonia oxidation, homologs of the heme-containing hydroxylamine oxidoreductase (*hao*) gene of ammonia-oxidizing bacteria (AOB) were absent from the AR1 and AR2 genomes, as in other AOA genomes [14],[15],[17],[25]. However, Vajrala et al. [36] observed hydroxylamine-induced oxygen consumption and ATP production in the marine ammonia-oxidizing archaeon *N*. *maritimus*. The number and sequences of six putative genes encoding copper-containing oxidases, which were suggested to function as possible hydroxylamine oxidoreductases (HAOs) [15], were conserved between *N*. *maritimus* and strains AR1 and AR2, encoding proteins with 88% amino acid identity on average. The number of putative genes encoding copper-containing oxidases found in the AOA genomes was six for *Ca*. “N. gargensis” and 3–4 for *Ca*. “Na. koreensis”, *Ca*. “Na. limnia”, and *Ca*. “C. symbiosum”. A putative gene for copper-containing oxidase was highly conserved (average 83% amino acid identity) between soil strain *Ca*. “Na. koreensis” (MY1_0289) and the marine AOA genomes (Nmar_1131, AR1_298, and AR2_318), and warrants further investigation as a possible HAO candidate. The other putative copper-containing oxidase gene, *nirK*, was highly conserved in all AOA, which might be involved in nitrifier denitrification [37]. A TATA box and parts of a BR element (transcription factor B recognition element), 23 nt or 25 nt upstream of the *nirK* gene ( Figure S9), were observed as in the archaeal *amo* gene [35], suggesting that the *nirK* gene could be expressed independently under the control of its own promoter.

As in other AOA genomes, strains AR1 and AR2 appear to encode a complete respiratory chain with complexes I–V, which are used for energy generation and reverse electron transport. The components have ∼93% amino acid identity to those of *N*. *maritimus*. Complex V is an archaeal type ATPase that is known to use both Na^+^ and proton gradients to generate ATP [38]. Na^+^ is frequently used instead of H^+^ in gradient formation during electron transport in oligotrophic or energy-stressed environments, since Na^+^ is usually less permeable to the cellular membrane.

Like other AOA genomes, the genomes of AR1 and AR2 lack homologs of cytochrome *c* proteins [15]–[17],[25], and therefore blue copper-containing proteins (Table S6) might be involved in the transfer of electrons from complex III. Known homologs encoding essential genes for heme biosynthesis (*ahb-nirJ1* and *ahb-nirJ2*) were missing [39] and putative genes for heme-containing proteins were rare in the AOA genomes. The only heme-containing gene detected in the AOA genomes (including AR1 and AR2) was that encoding the cytochrome b/b6 family protein of respiratory complex III. Since heme uptake by prokaryotes from the environment is not plausible [40], AOA genomes require further screening and analysis to characterize gene sets for heme biosynthesis. The variability in iron availability in marine and terrestrial environments suggests that the abundance of copper-containing oxidases for redox reactions in both soil (e.g., *Ca*. “Na. koreensis”) and marine AOA might be an evolutionary trait of *Thaumarchaeota* rather than a functional or environmental adaptation of the AOA. The high abundance of multicopper-containing proteins and blue copper-containing proteins in AOA, rather than heme-containing proteins, implies that ammonia oxidation pathways and respiratory chains in AOA groups I.1a and I.1b may be novel and conserved.

#### Carbon fixation

Most AOA characterized to date are able to grow chemolithotrophically using inorganic carbon (carbon dioxide and/or bicarbonate) as their sole carbon source [4],[7],[9]–[11],[22]. By contrast with their bacterial counterparts, AOA genomes do not contain key genes for the Calvin-Bassham-Benson cycle [41],[42], but might instead utilize the 3-hydroxtpropionate/4-hydroxybutyrate pathway. The genes encoding the three main proteins for this pathway, 4-hydroxybutyrate-CoA dehydratase, acetyl-CoA carboxylase, and methylmalonyl-CoA epimerase, were present in the AR1 and AR2 genomes and the putative proteins had 80–98% amino acid identity to the *N*. *maritimus* homologs.

Stable-isotopic and molecular studies raised questions regarding the mixotrophic nature of the marine lineage of group I.1a [43],[44]. Ammonia oxidation and growth of *N*. *viennensis* (a soil lineage of group I.1b) was supported by pyruvate and some pyruvate carbons were incorporated into archaeal cells [9]. Genes encoding proteins that are possibly involved in the transport of organic compounds, such as carbohydrates, amino acids, oligo/dipeptides, and nucleosides, were evident in the AR1 and AR2 genomes and in other AOA genomes. However, there has been no direct biochemical and physiological evidence from cultivated AOA to support the hypothesis that the marine lineage of group I.1a is mixotrophic. The *Ca*. “N. gargensis” genome encodes alanine dehydrogenase and an array of pyruvate transformation genes [25], suggesting that *Ca*. “N. gargensis” might utilize pyruvate or alanine as an alternative carbon source, by contrast with other AOA. Pyruvate phosphate dikinase, which is involved in the transformation of pyruvate to phosphoenolpyruvate for gluconeogenesis, was encoded in the genomes of marine AOA, including the AR1 and AR2 strains.

### Genomic traits of the deep marine sedimentary AOA

#### Urea utilization

**Figure 2:**
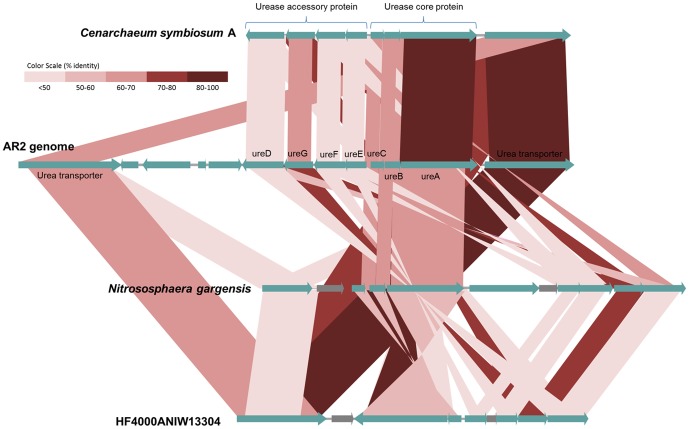
Comparison of the *Ca*. “Nitrosopumilus sediminis” AR2 genomic region containing genes for urea utilization with those of *Ca*. “Cenarchaeum symbiosum” and environmental metagenomes. *Ca*. “N. sediminis” AR2 genome is central, with the *Ca*. “C. symbiosum”, *Ca*. “Nitrososphaera gargensis”, and environmental metagenomic regions above and below, respectively. Homologous genes are connected with shaded regions, and the shaded color indicates the percent identity as determined by TBLASTX.

A complete set of genes involved in urea utilization was identified in the AR2 genome (Figure 2). This was absent from other marine (AR1 and *N*. *maritimus*) and soil/low-salinity AOA (*Ca*. “Na. koreensis” and *Ca*. “Na. limnia”) genomes. Urease operons were identified in the genomes of *Ca*. “C. symbiosum” [14], *N*. *viennensis* [45], *Ca*. “N. salaria” [19] and *Ca*. “N. gargensis” [25], and in a scaffold from a recent ocean metagenomic study [18], with 46–86% amino acid identities to the AR2 operon, respectively. Moreover, two copies of a urea transporter gene were identified in the AR2 genome that were 50–76% identical to the *dur3* gene from *Ca*. “C. symbiosum”, *Ca*. “N. gargensis”, and to the *dur3* gene from the Pacific Ocean metagenome recovered from a 4,000 m depth at station ALOHA [46]. A recruitment analysis comparing the AR2 genome to a Sargasso Sea metagenome showed that the archaeal urease utilization trait was widespread in water-column archaea. Since urea comprises a significant proportion of the dissolved nitrogen compounds in the surface layer of marine sediment [47], the capacity for urea utilization within sedimentary AOA may confer a selective advantage within that niche. Moreover, Alonso-Sáez et al. [48] suggested that deep water *Thaumarchaeota* in the Arctic and Antarctic oceans use urea as an energy source in nitrification.

#### Ectoine synthesis

Ectoine is a compatible solute that is found in a wide range of bacteria. The AR1 and AR2 genomes (as well as that of *N*. *maritimus* [49]) contained all four genes in the archaeal ectoine biosynthesis cluster (*ectA*, *ectB*, *ectC*, and *ectD*). In AR1 and AR2, the ectoine gene clusters were located in the centers of GI 6 and GI 3, respectively and the codon usage in these islands deviated markedly from the conserved core genes in the AR genome (Table S3). Recruitment analysis did not find ectoine biosynthesis genes in the Sargasso Sea metagenome or the *Ca*. “Na. limnia”, *Ca*. “Na. koreensis”, *Ca*. “N. gargensis”, or *Ca*. “C. symbiosum” genomes [13],[16],[17],[25]. Instead, *Ca*. “Na. limnia”, *Ca*. “Na. koreensis”, and *Ca*. “N. gargensis” employ mechanosensitive ion channels (MS channels; *mscS* and *mscL* genes) for regulating osmotic pressure. The AR1, AR2, and *N*. *maritimus* genomes also harbored genes for a small-conductance MS channel (*mscS*), but no large-conductance MS channel gene (*mscL*) was apparent; thus the ability to synthesize ectoine might be an important osmotic adaptation in members of the genus *Nitrosopumilus*.

#### Clustered regularly interspaced short palindromic repeats (CRISPRs)/Cas system

The CRISPR/Cas system mediates resistance against phages, and is found in the majority of investigated *Archaea* genomes [50]. Possible spacer-repeat arrays were identified in the AR1 (n = 3) and AR2 (n = 1) genomes, but only a single CDS exhibited similarity to a gene encoding a Cas protein (CAS1-like) (see GI 4 and 6, respectively, in Table S3). It is unclear whether the putative CRISPR spacers observed in AR1 and AR2 are artifacts or instead represent remnants of previous CRIPSR-loci. By contrast with the wide distribution of CRISPR in archaea, only one thaumarchaeon (*Ca*. “N. gargensis”) has so far been found to contain a CRISPR-locus and associated CAS-genes [25].

#### Phosphate assimilation

High-affinity phosphate uptake genes are often found in AOA, including the recently published *Ca*. “N. gargensis” genome [25], but we were unable to identify a high-affinity, high-activity phosphate uptake operon (*pstSCAB*) in either of the AR1 or AR2 genomes. The absence of these genes in the deep marine sedimentary AOA metagenome datasets may reflect habitat-specific circumstances. It is likely that sufficient phosphate is available in marine sediment as phosphate levels up to 100 µM were previously noted [51]; this is 50-fold higher than phosphate concentrations in the marine water column (∼2.0 µM) [52].

#### Chlorite degradation

Perchlorate (ClO_4_^−^), chlorate (ClO_3_^−^) and chlorite (ClO_2_^−^) are important pollutants in groundwater, surface waters, and soils [53]. Several (per)chlorate-reducing bacteria, including *Dechloromonas aromatic, Idenella dechloratnas,* and nitrite-oxidizing bacteria [54], contain a *cld* gene, which encodes enzymes that degrade chlorite (ClO_2_^−^) to chloride (Cl^−^) and oxygen (O_2_). Although *cld* genes are not present in AOB genomes, they are contained in all AOA genomes examined to date, including the AR1, AR2, *N*. *maritimus*, *Ca*. “Na. koreensis”, *Ca*. “Na. limnia”, *Ca*. “N. gargensis”, and *Ca*. “C. symbiosum” genomes. Cld proteins in AR1 and AR2 exhibited 35–68% and 50–87% identity, respectively, with those of other AOA. Cld in AOA may be necessary for chlorite detoxification, since chlorite is a selective inhibitor of ammonia oxidation [55]. This concurs with our previous results [7],[10] showing that group I.1a AOA tolerated higher concentrations of chlorite than *Nitrosomonas europaea* [7],[10],[55].

### Genomic features of co-cultured SOB

Successful cultivation of sedimentary AOA reportedly depends upon co-cultivation with SOBs [7]. Epsilonproteobacterial and gammaproteobacterial genomes were major constituents of the AR and SJ culture sequences, as detailed herein. Because the metagenomic features of the *Epsilonproteobacteria* (cluster 2) and *Gammaproteobacteria* (cluster 3) from the AR and SJ cultures were nearly identical (reciprocal ANI 99%), we selected epsilonproteobacterial (cluster 2) and gammaproteobacterial (cluster 3) bins from the AR and SJ cultures, respectively, for further analysis. These are designated “EP_AR” and “GM_SJ”, and their metabolic capabilities as determined by genomic analysis are discussed below and summarized in Table S4.

Strain EP_AR was affiliated with chemolithoautotrophic SOB. Several key enzymes involved in sulfur oxidation (e.g., sulfur-compounds oxidation system, SOX) were encoded within the EP_AR genome [56] (Table S4). The putative SOX proteins had 55–92% amino acid identity to those of the close relatives *Sulfurovum* sp. NBC37-1 [24] and *Sulfurimonas denitrificans* DSM 1251 [57]. Strain GM_SJ resembled a typical marine heterotroph since no genes related to sulfur oxidation or carbon fixation were observed in the genome (Table S4).

Microbial interactions play a critical role in shaping niches for microorganisms in natural environments. Sedimentary AOA and SOB occupy similar niches in sediment redox gradients [58], since AOA and SOB at oxic-anoxic interfaces consume ammonia and sulfide, respectively, diffused from the anoxic layers of marine sediment. Joye and Hollibaugh [59] reported that sulfide (<100 µM) inhibits nitrification in marine sediments. The prevalence of AOA may therefore be assisted by SOB detoxification of sulfides. The unusually tight associations between AOA and SOB were described in a terrestrial cold sulfidic spring [60], and thaumarchaeotal strains were physically associated with SOB in sulfide-rich mangrove swamps [61]. Sulfide-quinone reductase (*sqr*), sulfite:cytochrome *c* oxidoreductase (*dsrAB*), and the SOX system genes (*soxYZABCFHL*) in the EP_AR genome could mediate sulfide oxidation reactions [62]. This suggests that strain EP_AR might be a natural co-habitant of sedimentary AOA, and, although we used thiosulfate instead of sulfide for enrichment in this study [7], interactions between SOB and AOA might be exploited for the successful enrichment of SJ and AR in the laboratory.

AOB have a low efficiency for N_2_O production during nitrifier denitrification and most NO is emitted to an extracellular environment [63],[64]. Excess NO is therefore potentially toxic to the nitrifier itself and to other bacteria. Nitric oxide is suggested as an intermediate during bacterial [65],[66] and archaeal nitrification. Archaeal NO production was suggested by genomic analysis [67] in this study and by Walker et al. [15] and is supported by the inhibition of AOA by NO scavengers [68]. N_2_O emissions during archaeal ammonia oxidation [69],[70] provide indirect evidence of the involvement of NO in archaeal nitrifier denitrification [10],[11]. A putative gene encoding toxic NO-detoxifying flavohemoglobin [NO dioxygenase, NOD, 51.4% amino acid identity with that in *Aquifex aeolicus* VF5 [71]] was observed in strain EP_AR ( Figure S10), while no homolog was found in the genome of the closest relative, *Sulfurovum* sp. NBC37-1 ( Table S4). A gene-encoding phage integrase [48% amino acid identity with that in *Sulfurimonas denitrificans* [57]] located upstream of the NOD gene suggests that the NOD gene may have been acquired through horizontal gene transfer. Catalytic NO dioxygenation occurs most effectively via NOD under aerobic conditions [72], while nitric oxide reductase would be active under anoxic conditions [73]. The NOD in co-cultured SOB might therefore play a role in stimulating AOA growth. Genomic analysis of co-cultured SOB suggested that sulfur and nitrogen metabolism might be involved in the interactions between sedimentary AOA and co-cultured bacteria. Further systematic investigations are warranted to determine the response of sedimentary AOA to nitric oxide scavengers and generators.

## Conclusions

Metagenomic analyses enabled the assembly of two distinct deep marine sediment-derived AOA genomes, AR1 and AR2, and the determination of genetic similarities and differences between these organisms and previously sequenced AOA. Many key genomic features were conserved between AR1 and AR2 and other AOA, including genes pertaining to energy metabolism and carbon fixation. Nevertheless, genomic variations were also apparent, including: 1) Large GIs comprising ∼15% of the total genomes were found in AR1 and AR2; 2) Approximately 24% of CDS in AR1 and AR2 were unique; and 3) High-affinity phosphate uptake genes were absent in AR1 and AR2. In addition, a urease operon was found in the AR2 genome, but not the AR1 genome, suggesting potentially distinctive strategies for resource utilization between the two deep marine sedimentary AOA strains.

The availability of the genome sequences of deep marine sedimentary AOA will provide a foundation for evolutionary, biochemical, and ecophysiological studies that will contribute to the understanding of niche adaptations in marine AOA.

## Materials and Methods

### Cultivation of sediment microorganisms and preparation and sequencing of metagenomic DNA

Details of the enrichment and properties of the AOA used for this study were described previously [7]. AOA were enriched from sediment samples collected from Donghae (128° 35_E, 38° 20_N; depth, 650 m) and Svalbard (Arctic region, 16° 28_E, 78 °21_N; depth, 78 m) and are referred to as SJ and AR cultures, respectively. The field studies did not involve endangered or protected species and no specific permits were required.

Ammonia (1 mM) and thiosulfate (0.1 mM) were used as energy sources and bicarbonate (3 mM) was used as a carbon source. The culture medium was supplemented with a trace element mixture and a vitamin solution. Ammonia consumption and nitrite production were monitored as described by Park et al. [7]. After the ammonia was exhausted, cultures were transferred to fresh medium (inoculum comprising 10% of total medium volume) and cultivated at 25°C in the dark. The culture was maintained by transferring a 10% inoculum to fresh culture medium approximately every 2 weeks. After 50 months, cells from a 1 L culture were harvested using 0.22 µm pore size filters (Millipore, Billerica, MA) with a vacuum pump. The filters were placed in a sterile conical tube and stored at −70°C. Total DNA was extracted using a modified method based on that described by Park et al. [74]. Briefly, filters were treated with DNA extraction buffer [75] at 60°C for 30 min, and nucleic acids were purified with phenol/chloroform/isoamyl alcohol and chloroform/isoamyl alcohol. Metagenomic DNA integrity was confirmed using 0.8% (w/v) agarose gel electrophoresis and DNA was quantified using a NanoDrop ND 1000 spectrophotometer. Total DNA (∼5 µg) was sequenced using single read and mate-paired (about 8 Kb insert library size) end sequencing methods using a 454 GS-FLX Titanium platform (Roche Applied Science, Indianapolis, IN). Sample sequencing and analytical data processing was performed at the National Instrumentation Center for Environmental Management, Seoul National University, South Korea. The average read length was approximately 291 bp for AR and 266 bp for SJ. Short sequences and sequences with a quality score <20 were removed to enhance metagenomic sequence quality.

### rRNA gene analysis

rRNA genes were identified by comparing the obtained datasets to the RDP database [76]. All reads that matched an rRNA sequence with an alignment length >100 bases and an e-value ≤ 0.001 were extracted. The best hit for each rRNA was used to assign a high taxonomic level (at or above class) to the sequence. Where possible, sequences were further assigned to a genus if they shared ≥ 95% rRNA sequence identity with rRNA from a known species.

### Assembly, annotation, and functional classification

Assembly was performed using the Roche GS De Novo Assembler (Newbler assembler v. 2.3, >98% identity and >40 bp overlap length). After assembly, putative CDS were predicted using MetaGeneAnnotator [77]. Protein sequences were annotated using the best BLAST hit against the NCBI NR database, and tRNAs were identified using tRNAscan-SE [78]. Entire metagenome datasets were annotated using the MG-RAST server [79].

Assembled contigs that were <5 kb in length and those with fewer than three predicted genes were discarded. Contigs were only retained that yielded consistent hits to a single high-level taxon (e.g., *Epsilonproteobacteria*, *Thaumarchaeota*, and *Gammaproteobacteria*). Strict assembly requirements combined with a taxonomic uniformity condition imposed on the assembled sequences resulted in 118 (in AR culture) and 91 (in SJ culture) contigs that were >5 Kb in length, had a consistent phylogenetic profile, and were likely to originate from a single organism (e.g., *Sulfurovum* sp. NBC37-1 and *N*. *maritimus*). To test if the assembly strategy produced contigs that were “real,” we manually identified all contigs that belonged to the clades of *Ca*. “Nitrosopumilus” and *Sulfurovum*, which were abundant in both enrichment cultures. The criterion for assigning contigs to the clades of *Ca*. “Nitrosopumilus” and *Sulfurovum* was that all genes must provide best hits in these genomes. We identified 97 contigs (73 for *Ca*. “Nitrosopumilus” and 24 for *Sulfurovum*) in which all genes provided the best hit for *N*. *maritimus* and *Sulfurovum* sp. NBC37-1. To increase taxonomic uniformity, we directly compared the nucleotide sequence of these contigs to the reference genome, using BLASTN [80],[81]. Oligonucleotide frequencies of the assembled contigs were computed using the wordfreq program in the EMBOSS package [82], and principal component analysis was performed using the R package FactoMineR [83]. All predicted CDS were also searched for similarity using RPSBLAST to predict clusters of orthologous group assignments (cutoff e-value of 10^−5^) [84]. We used CUSP and CODCMP from the European Molecular Biology Open Software Suite package for codon usage analysis. The GC skew was calculated using the Oligoweb interface http://insilico.ehu.es/oligoweb/. CRISPRs were searched using CRISPR Finder [85].

### Metagenomic comparisons

Reciprocal BLASTN and TBLASTX searches between the metagenomes were used for comparative analyses, leading to the identification of regions of similarity, insertions, and/or rearrangements (e-value cutoff of 10^−5^). The Artemis Comparison Tool [86] was used to visualize comparisons of the genomic fragments. ANI was calculated as defined by Konstantinidis and Tiedje [26]. Reciprocal BLASTCLUST was used to predict orthologous proteins between each contig (affiliated with *Thaumarchaeota*, *Epsilonproteobacteria*, and *Gammaproteobacteria*) and reference genome (e.g., *N*. *maritimus* and *Sulfurovum* sp. NBC37-1) using a minimum cutoff of 50% identity and 70% of the length of the query CDS. The JSpecies program [87] was used to confirm manual ANI analyses. A BLASTN [88] comparison (cutoff of 50% identity and 70% of the length of the query sequences) between the datasets formed by the two archaeal genomes and the metagenome dataset of the Sargasso Sea [3] was used for recruitment analysis.

### Accession numbers

Sequence data are deposited in Genbank under the following BioProject IDs: PRJNA66411, PRJNA66413, and PRJDA162597.

## Supporting Information

Figure S1 GC content (%) of single and mate-paired reads of the AR and SJ metagenomes. The numbers of single reads of the AR and SJ metagenomes were about 727,301 and 631,686, and of mate-paired reads of AR and SJ metagenomes were 478,179 and 489,454, respectively.
(TIF)

Figure S2 Taxonomic profiles (at or above class level) using the 16S rRNA gene sequences of (A) AR (n  =  1,100) and (B) SJ (n  =  908) metagenome datasets.
(TIF)

Figure S3 Comparison of all sequence reads from the AR and SJ metagenome datasets with the M4N5 database using the MG-RAST server (BLASTX cutoff: e-value of 1e-5 and minimum alignment length of 50 bp).
(TIF)

Figure S4 GC% versus length of assembled contigs (≥5 Kb) from the AR (A) and SJ (B) metagenomes.
(TIF)

Figure S5 Phylogenetic analysis of the archaeal 16S rRNA gene sequences obtained from strain AR1 and AR2 indicated in boldface and published sequences. “ThAOA” indicates thermophilic AOA lineage. Cluster groups were denoted at the right of the figure based on the origin of reference sequences. Branching patterns supported by more than 50% bootstrap values (1,000 iterations) by means of neighbor-joining was denoted by their respective bootstrap values. The scale bar represents 2% estimated sequence divergence.
(TIF)

Figure S6 Dot plot representation of the pairwise alignments of the strain AR1 and SCM1 (A), AR2 and SCM1 (B), and AR1 and AR2 (C) genomes. Alignments were performed on the six-frame amino acid translation of the genome sequences using the program in the MUMmer 3.23 package. In all plots, a dot indicates a gene compared, with forward or reverse matches shown in red and blue, respectively.
(TIF)

Figure S7 Recruitment plots of the Sargasso Sea metagenome dataset of GOS to the draft genomes of (A) *Ca*. “Nitrosopumilus koreensis” AR1 and (B) *Ca*. “N. sediminis” AR2. (1) GC-content plotted with a sliding window of 25,000 nucleotides. Average percentage of GC (34.2% and 33.6%, respectively) is shown by red line. (2) GC skew of AR1 and AR2 draft genomes plotted with a sliding window of 25,000 nucleotides. (3) Mummerplot showing recruitment of the Sargasso Sea metagenome reads to the AR1 and AR2 draft genomes. Individual archaeal reads of the metagenome were blasted with the AR1 and AR2 draft genomes, respectively. Green boxes indicate genomic islands of the AR1 and AR2 draft genomes.
(TIF)

Figure S8 Distribution of COG functional classes. Percentage of COGs predicted in the *Ca*. “Nitrosopumilus koreensis” AR1 and *Ca*. “N. sediminis” AR2 genomes. All genes of both genomes (A) and genes found in genomic islands (B). COG; cluster of orthologous groups.
(TIF)

Figure S9 Alignment of start and upstream region of the nirK gene sequence from metagenome and cultivated marine ammonia-oxidizing archaea. The ATG start codon and TATAbox/Brelements are highlighted [2]. NirK gene sequences are from *Nitrosopumilus maritimus* (nmar), *N*. *koreensis* (ar1), *N*. *sediminis* (ar2) and marine metagenome (Marine-met).
(TIF)

Figure S10 Phylogenetic analysis of the NO dioxygenase gene in strain EP_AR indicated in boldface and homolog enzymes based on amino acid sequences. “EUK” indicates *Eukaryote* domain. Branching patterns supported by more than 50% bootstrap values (1,000 iterations) by means of neighbor-joining was denoted by their respective bootstrap values. The scale bar represents 20% estimated sequence divergence.
(TIF)

Table S1 General features of the metagenome datasets from the AR and SJ cultures.
(DOCX)

Table S2 Nucleotide (NT) and amino acid (AA) identities of rRNA and ammonia monooxygenase (*amo*) genes, respectively between archaeal genomes (AR1, AR2, and SJ) and *Nitrosopumilus maritimus*.
(DOCX)

Table S3 Characteristics of genomic islands of the draft genomes of *Ca*. “Nitrosopumilus koreensis” AR1 and *Ca*. “N. sediminis” AR2.
(DOCX)

Table S4 Summary of predicted metabolic capabilities of microorganisms based on draft genome sequences.
(XLSX)

Table S5 Putative coding sequences of the genomic islands of the AR1 and AR2 genomes.
(XLSX)

Table S6 Comparison of genes coding blue copper domain-containing carriers and thiol-disulfide oxidoreductase between *Ca*. “Nitrosopumilus koreensis” AR1 and *Ca*. “N. sediminis” AR2, and *N*. *maritimus* genomes.
(DOCX)
