# Supplementary figures and images for: Genomes of Two New Ammonia-Oxidizing Archaea Enriched from Deep Marine Sediments
Source: PLoS One. 2014 May 5;9(5):e96449. doi: 10.1371/journal.pone.0096449 (PMC4010524; doi:10.1371/journal.pone.0096449)

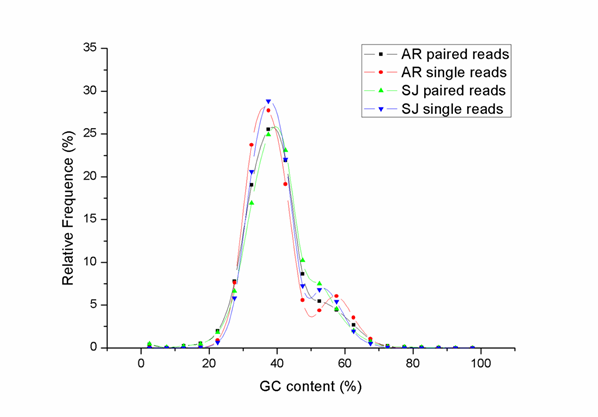

Supplement: Figure S1 — GC content (%) of single and mate-paired reads of the AR and SJ metagenomes. The numbers of single reads of the AR and SJ metagenomes were about 727,301 and 631,686, and of mate-paired reads of AR and SJ metagenomes were 478,179 and 489,454, respectively. (TIF) [file pone.0096449.s001.tif]

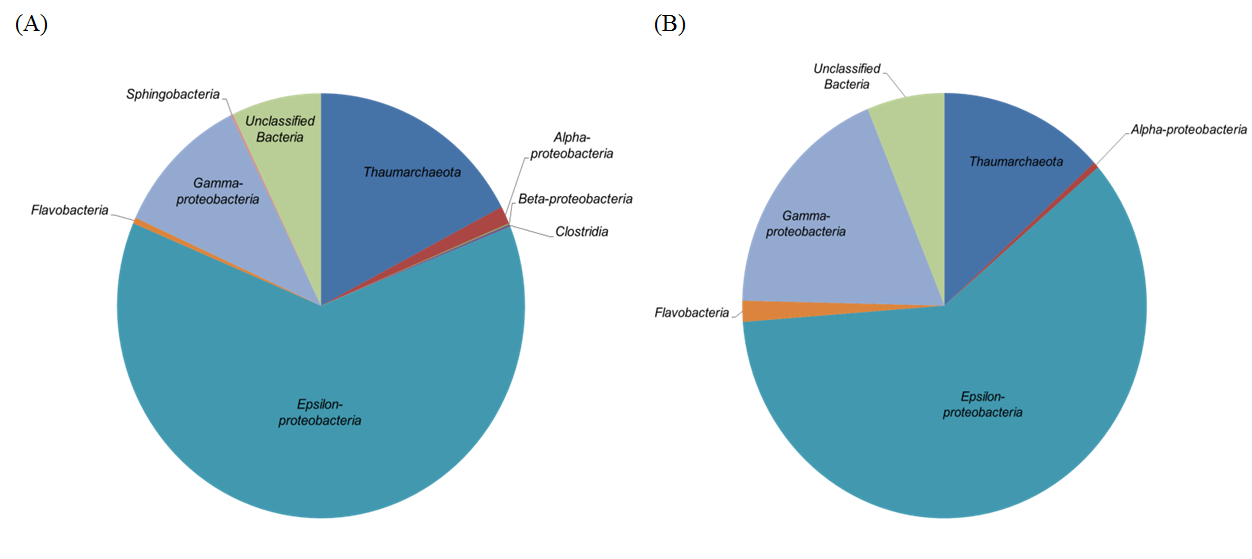

Supplement: Figure S2 — Taxonomic profiles (at or above class level) using the 16S rRNA gene sequences of (A) AR (n = 1,100) and (B) SJ (n = 908) metagenome datasets. (TIF) [file pone.0096449.s002.tif]

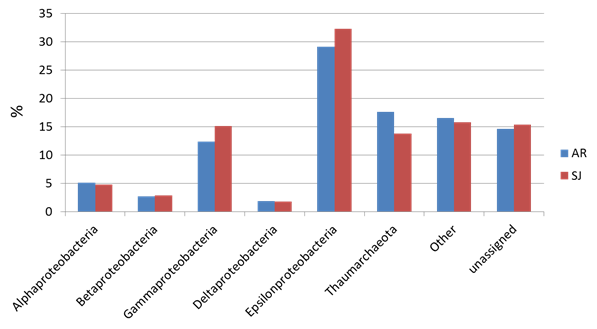

Supplement: Figure S3 — Comparison of all sequence reads from the AR and SJ metagenome datasets with the M4N5 database using the MG-RAST server (BLASTX cutoff: e-value of 1e-5 and minimum alignment length of 50 bp). (TIF) [file pone.0096449.s003.tif]

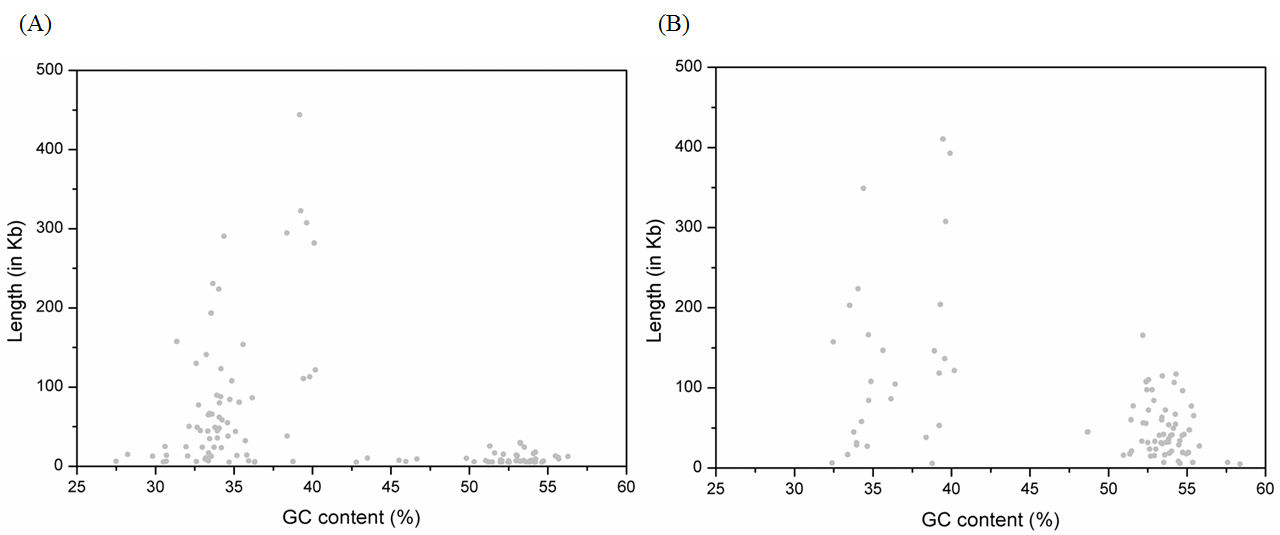

Supplement: Figure S4 — GC% versus length of assembled contigs (≥5 Kb) from the AR (A) and SJ (B) metagenomes. (TIF) [file pone.0096449.s004.tif]

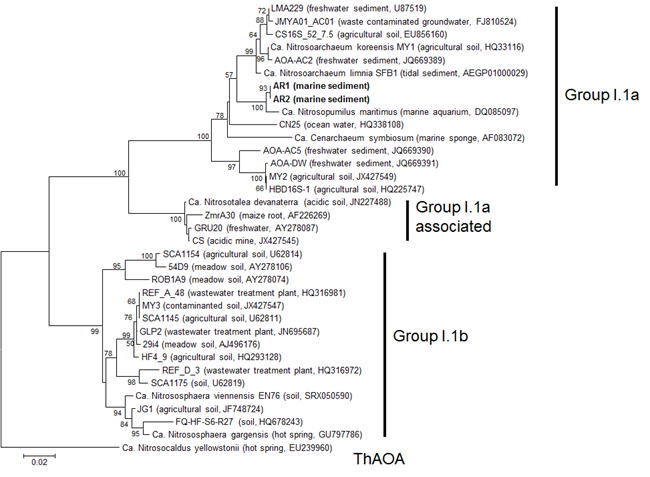

Supplement: Figure S5 — Phylogenetic analysis of the archaeal 16S rRNA gene sequences obtained from strain AR1 and AR2 indicated in boldface and published sequences. “ThAOA” indicates thermophilic AOA lineage. Cluster groups were denoted at the right of the figure based on the origin of reference sequences. Branching patterns supported by more than 50% bootstrap values (1,000 iterations) by means of neighbor-joining was denoted by their respective bootstrap values. The scale bar represents 2% estimated sequence divergence. (TIF) [file pone.0096449.s005.tif]

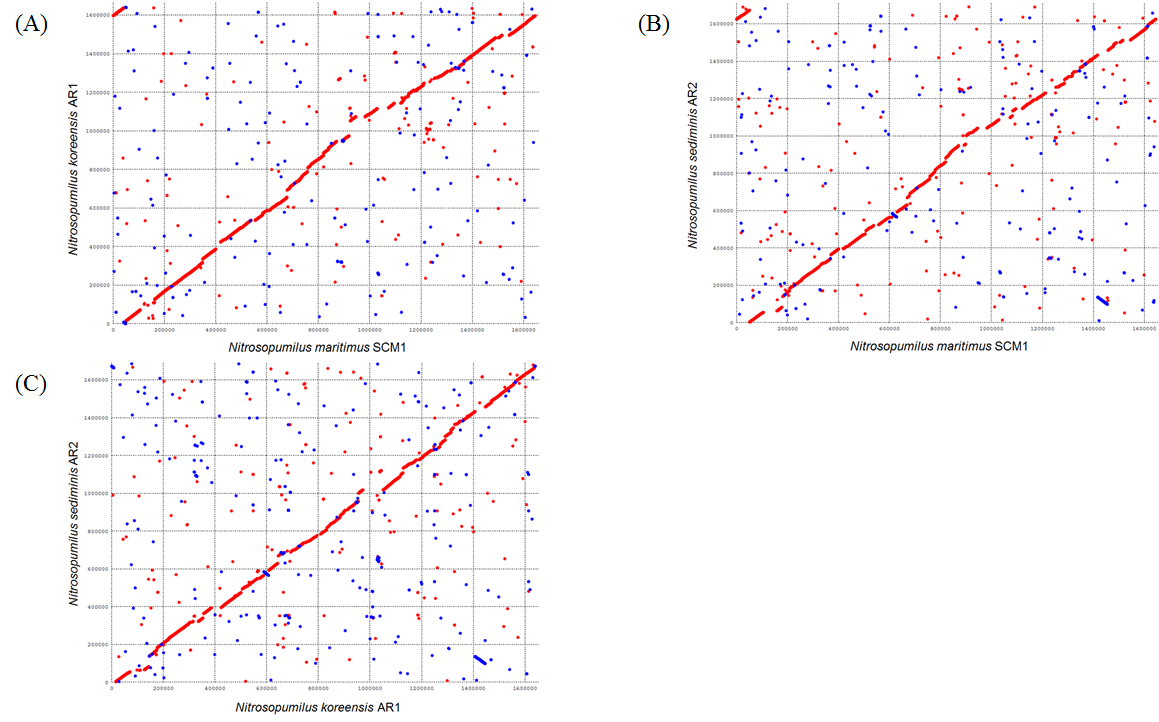

Supplement: Figure S6 — Dot plot representation of the pairwise alignments of the strain AR1 and SCM1 (A), AR2 and SCM1 (B), and AR1 and AR2 (C) genomes. Alignments were performed on the six-frame amino acid translation of the genome sequences using the program in the MUMmer 3.23 package. In all plots, a dot indicates a gene compared, with forward or reverse matches shown in red and blue, respectively. (TIF) [file pone.0096449.s006.tif]

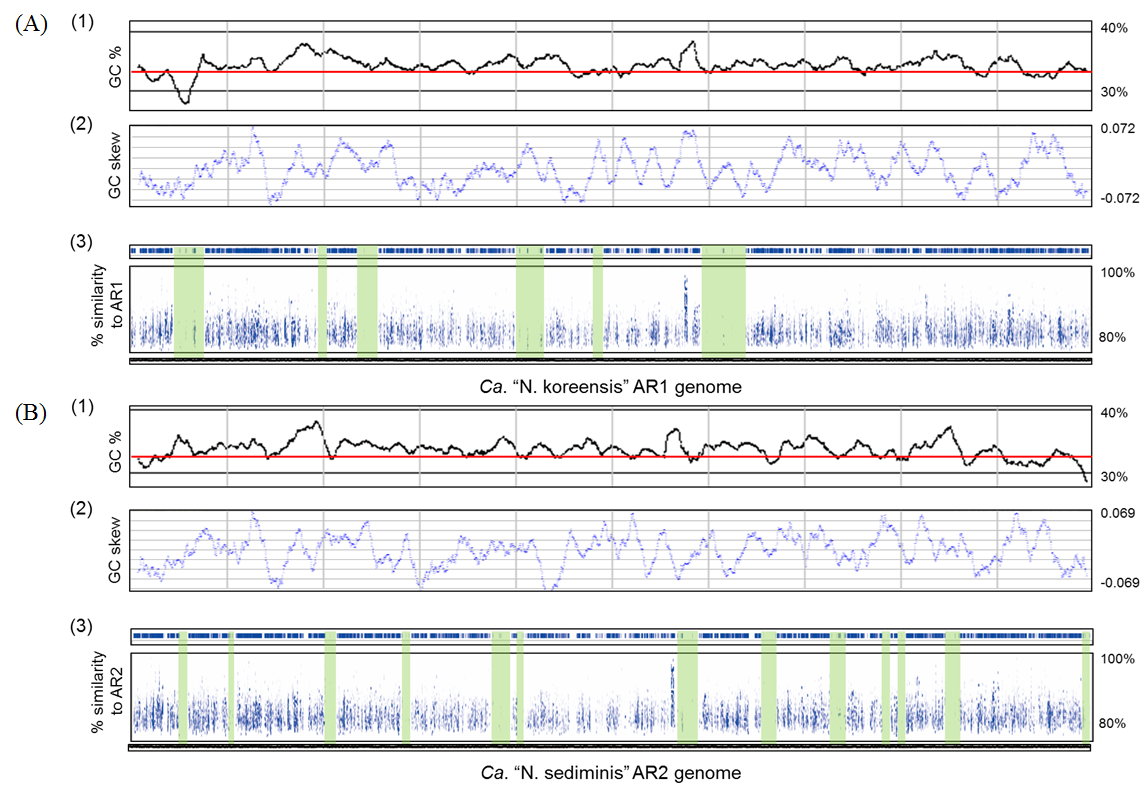

Supplement: Figure S7 — Recruitment plots of the Sargasso Sea metagenome dataset of GOS to the draft genomes of (A) Ca. “Nitrosopumilus koreensis” AR1 and (B) Ca. “N. sediminis” AR2. (1) GC-content plotted with a sliding window of 25,000 nucleotides. Average percentage of GC (34.2% and 33.6%, respectively) is shown by red line. (2) GC skew of AR1 and AR2 draft genomes plotted with a sliding window of 25,000 nucleotides. (3) Mummerplot showing recruitment of the Sargasso Sea metagenome reads to the AR1 and AR2 draft genomes. Individual archaeal reads of the metagenome were blasted with the AR1 and AR2 draft genomes, respectively. Green boxes indicate genomic islands of the AR1 and AR2 draft genomes. (TIF) [file pone.0096449.s007.tif]

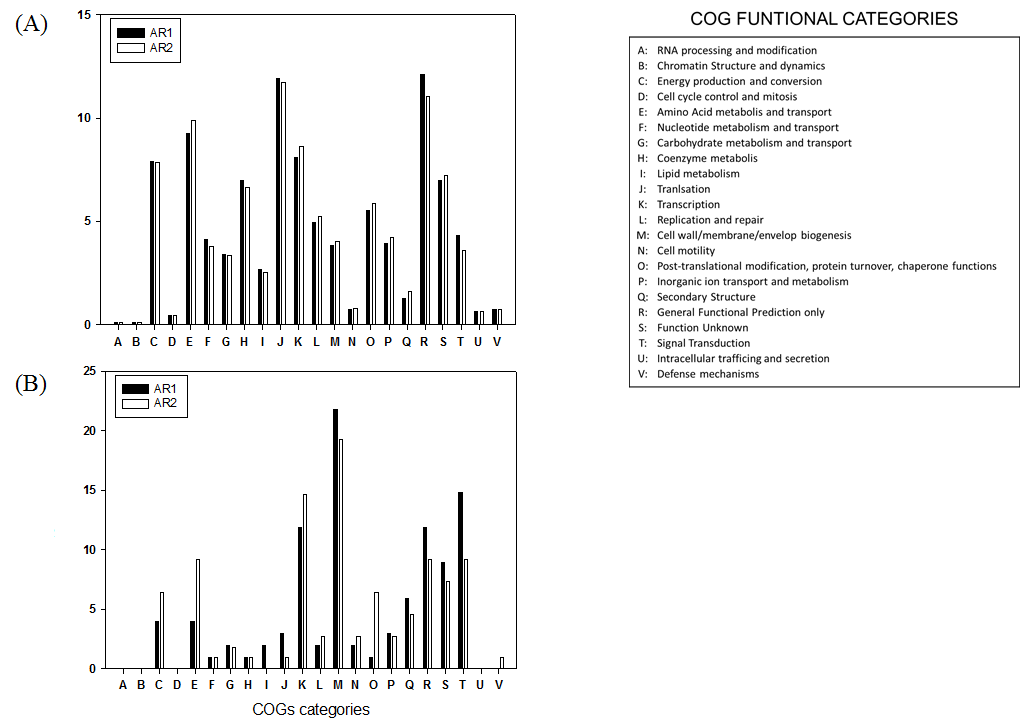

Supplement: Figure S8 — Distribution of COG functional classes. Percentage of COGs predicted in the Ca. “Nitrosopumilus koreensis” AR1 and Ca. “N. sediminis” AR2 genomes. All genes of both genomes (A) and genes found in genomic islands (B). COG; cluster of orthologous groups. (TIF) [file pone.0096449.s008.tif]

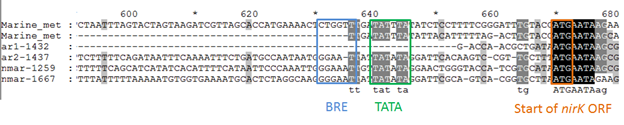

Supplement: Figure S9 — Alignment of start and upstream region of the nirK gene sequence from metagenome and cultivated marine ammonia-oxidizing archaea. The ATG start codon and TATAbox/Brelements are highlighted [2]. NirK gene sequences are from Nitrosopumilus maritimus (nmar), N. koreensis (ar1), N. sediminis (ar2) and marine metagenome (Marine-met). (TIF) [file pone.0096449.s009.tif]

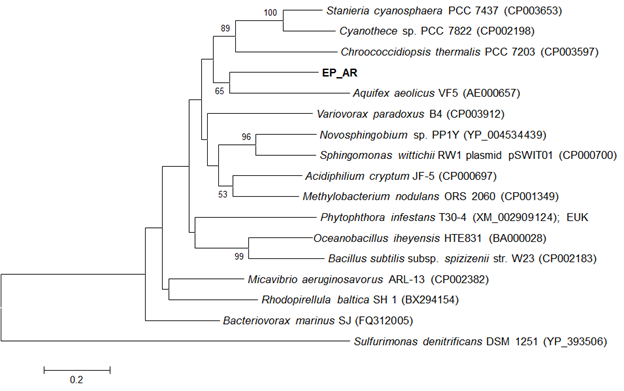

Supplement: Figure S10 — Phylogenetic analysis of the NO dioxygenase gene in strain EP_AR indicated in boldface and homolog enzymes based on amino acid sequences. “EUK” indicates Eukaryote domain. Branching patterns supported by more than 50% bootstrap values (1,000 iterations) by means of neighbor-joining was denoted by their respective bootstrap values. The scale bar represents 20% estimated sequence divergence. (TIF) [file pone.0096449.s010.tif]
