# Supplementary material for: Genomes of Two New Ammonia-Oxidizing Archaea Enriched from Deep Marine Sediments
Source: PLoS One. 2014 May 5;9(5):e96449. doi: 10.1371/journal.pone.0096449 (PMC4010524; doi:10.1371/journal.pone.0096449)
Supplement: Table S1 — General features of the metagenome datasets from the AR and SJ cultures. (DOCX) [file pone.0096449.s011.docx]

**Table S1**. General features of the metagenome datasets from the AR and SJ cultures.

| **Datasets** | **AR** | **SJ** |
| --- | --- | --- |
| **Sequencing Methods** | Single end & Mate-paired end | Single end & Mate-paired end |
| **Number of Reads** | 1,573,228 | 901,925 |
| **Dataset Size (bp)** | 536,810,491 | 308,163,881 |
| **Average Read Length (bp)** | 291 | 266 |
| **Number of Contigs** | 15,155 | 2,595 |
| **Largest Contig Size (bp)** | 443,878 | 410,547 |
| **Average GC (%) of Contigs** | 45.9 | 43.7 |
