# Supplementary material for: Genomes of Two New Ammonia-Oxidizing Archaea Enriched from Deep Marine Sediments
Source: PLoS One. 2014 May 5;9(5):e96449. doi: 10.1371/journal.pone.0096449 (PMC4010524; doi:10.1371/journal.pone.0096449)
Supplement: Table S2 — Nucleotide (NT) and amino acid (AA) identities of rRNA and ammonia monooxygenase (amo) genes, respectively between archaeal genomes (AR1, AR2, and SJ) and Nitrosopumilus maritimus. (DOCX) [file pone.0096449.s012.docx]

**Table S2**. Nucleotide (NT) and amino acid (AA) identities of rRNA and ammonia monooxygenase (*amo*) genes, respectively between archaeal genomes (AR1, AR2, and SJ) and *Nitrosopumilus* *maritimus*.

|  |  | AR1 to | | | AR2 to | SJ to | |
| --- | --- | --- | --- | --- | --- | --- | --- |
|  |  | SJ | AR2 | *N*. *maritimus* | *N*. *maritimus* | AR2 | *N*. *maritimus* |
| rRNA |  |  |  |  |  |  |  |
| 23S | NT | 99.8 | 96.6 | 98.8 | 96.6 | 96.6 | 98.9 |
| 16S | NT | 99.2 | 99.7 | 99.1 | 99 | 99.1 | 99.8 |
| 5S | NT | 100 | 96.5 | 98.6 | 95.1 | 96.5 | 98.6 |
| *amo* |  |  |  |  |  |  |  |
| *amoA* | NT | 100 | 93.8 | 95.6 | 93.2 | 93.8 | 95.6 |
|  | AA | 100 | 98.1 | 99.3 | 98.7 | 98.1 | 99.3 |
| *amoB* | NT | 100 | 91.5 | 96.3 | 90.0 | 91.5 | 96.3 |
|  | AA | 100 | 95.7 | 97.3 | 93.1 | 95.7 | 97.3 |
| *amoC* | NT | 100 | 92.6 | 97.1 | 90.2 | 91.3 | 98.5 |
|  | AA | 100 | 98.7 | 100 | 95.7 | 95.2 | 98.4 |
