# Supplementary material for: Genomes of Two New Ammonia-Oxidizing Archaea Enriched from Deep Marine Sediments
Source: PLoS One. 2014 May 5;9(5):e96449. doi: 10.1371/journal.pone.0096449 (PMC4010524; doi:10.1371/journal.pone.0096449)
Supplement: Table S3 — Characteristics of genomic islands of the draft genomes of Ca. “Nitrosopumilus koreensis” AR1 and Ca. “N. sediminis” AR2. (DOCX) [file pone.0096449.s013.docx]

**Table S3**. Characteristics of genomic islands of the draft genomes of *Ca*. “Nitrosopumilus koreensis” AR1 and *Ca*. “N. sediminis” AR2.

| **GI NO.** | **Start** | **End** | **Length  (bp)** | **Number of genes** | **GC  content (%)** | **Hypothetical  proteins** | **GOS**  **recruitment** | **Inferred  character** | **Codon  usage  deviation^*^** |
| --- | --- | --- | --- | --- | --- | --- | --- | --- | --- |
| ***Ca*. “N. koreensis” AR1** | | | | | | | | | |
| 1 | 70,865 | 125,801 | 54,937 | 69 | 31 | 26 | - | Cell wall synthesis | 3.740 |
| 2 | 316,357 | 335,395 | 19,039 | 24 | 36 | 15 | - | Antibiotic Resistance | 6.409 |
| 3 | 386,875 | 423,228 | 36,354 | 42 | 34 | 26 | - | Putative phage  /Antibiotic resistance | 4.560 |
| 4 | 655,435 | 708,874 | 53,440 | 89 | 35 | 48 | - | Putative phage (CRISPR/Cas) | 4.085 |
| 5 | 790,075 | 808,096 | 18,022 | 17 | 33 | 8 | - | - | 5.369 |
| 6 | 973,387 | 1,050,998 | 77,612 | 106 | 34 | 52 | - | Osmotic stress tolerance  /Putative phage | 4.193  (7.470)^**^ |
| ***Ca*. “N. sediminis” AR2** | | | | | | | | | |
| 1 | 83,908 | 98,924 | 15,017 | 20 | 37 | 12 | - | Putative cell wall synthesis | 5.683 |
| 2 | 173,007 | 182,294 | 9,288 | 15 | 32 | 8 | - | - | 3.816 |
| 3 | 340,775 | 363,426 | 22,652 | 41 | 32 | 24 | - | Osmotic stress tolerance | 3.825  (4.514)^**^ |
| 4 | 473,344 | 491,875 | 18,532 | 36 | 33 | 17 | - | Chaperon | 5.132 |
| 5 | 630,923 | 668,212 | 37,290 | 46 | 35 | 34 | - | - | 4.921 |
| 6 | 678,545 | 694,869 | 16,325 | 30 | 32 | 13 | - | Putative phage (CRISPR/Cas) | 4.298 |
| 7 | 956,494 | 1,005,494 | 49,001 | 66 | 33 | 41 | - | Secretion system | 3.271 |
| 8 | 1,107,585 | 1,134,934 | 27,350 | 38 | 31 | 23 | - | Signal transduction | 3.617 |
| 9 | 1,348,317 | 1,362,626 | 14,310 | 25 | 31 | 12 | - | - | 3.047 |
| 10 | 1,432,373 | 1,458,500 | 26,128 | 26 | 35 | 4 | - | Cell wall synthesis | 6.671 |
| 11 | 1,321,523 | 1,333,795 | 12,273 | 15 | 33 | 3 | - | - | 4.700 |
| 12 | 1,669,692 | 1,690,585 | 20,894 | 23 | 28 | 6 | - | Cell wall synthesis | 1.917 |

^*^Differences in codon usage within an island and the entire genome [[1](#_ENREF_1)].

^**^Value within operon of ectoine synthesis and the entire genome.

**Supplementary references**

1. Ivars-Martinez E, Martin-Cuadrado AB, D'Auria G, Mira A, Ferriera S, et al. (2008) Comparative genomics of two ecotypes of the marine planktonic copiotroph *Alteromonas macleodii* suggests alternative lifestyles associated with different kinds of particulate organic matter. ISME J 2: 1194-1212.

2. Bell SD, Kosa PL, Sigler PB, Jackson SP (1999) Orientation of the transcription preinitiation complex in archaea. Proc Natl Acad Sci U S A 96: 13662-13667.
