# Supplementary material for: Genomes of Two New Ammonia-Oxidizing Archaea Enriched from Deep Marine Sediments
Source: PLoS One. 2014 May 5;9(5):e96449. doi: 10.1371/journal.pone.0096449 (PMC4010524; doi:10.1371/journal.pone.0096449)
Supplement: Table S6 — Comparison of genes coding blue copper domain-containing carriers and thiol-disulfide oxidoreductase between Ca. “Nitrosopumilus koreensis” AR1 and Ca. “N. sediminis” AR2, and N. maritimus genomes. (DOCX) [file pone.0096449.s016.docx]

**Table S6.** Comparison of genes coding blue copper domain-containing carriers and thiol-disulfide oxidoreductase between *Ca*. “Nitrosopumilus koreensis” AR1 and *Ca*. “N. sediminis” AR2, and *N*. *maritimus* genomes.

| **Blue (type 1) copper domain protein** | | | |
| --- | --- | --- | --- |
| *N*. *maritimus* | Annotation | Similarity (%) to | |
|  |  | AR1 | AR2 |
| Nmar_0815 | soluble periplasmic BCP | 77 (AR1_145)* | 85 (AR2_81) |
| Nmar_1102 | soluble periplasmic BCP | 82 (AR1_1309) | 65 (AR2_184) |
| Nmar_1443 | soluble periplasmic BCP | 88 (AR1_1569) | - |
| Nmar_1637 | soluble periplasmic BCP | -** | - |
| Nmar_0004 | soluble periplasmic BCP | - | - |
| Nmar_1307 | soluble periplasmic BCP | 89 (AR1_1465) | 85 (AR2_1472) |
| Nmar_0918 | periplasmic membrane BCP | - | - |
| Nmar_1678 | periplasmic membrane BCP |  |  |
| Nmar_1273 | periplasmic membrane BCP | - | - |
| Nmar_1161 | periplasmic membrane BCP | - | - |
| Nmar_1226 | periplasmic membrane BCP | 94 (AR1_1377) | 87 (AR2_1399) |
| Nmar_1142 | cytoplasmic membrane BCP | 93 (AR1_1668) | 90 (AR2_1729) |
| Nmar_1542 | cytoplasmic membrane BCP | - | - |
| Nmar_0185 | periplasmic membrane BCP | - | - |
| Nmar_1665 | soluble periplasmic BCP | - | - |
| Nmar_1129 | cytoplasmic membrane BCP | - | - |
| Nmar_1650 | periplasmic membrane BCP |  | - |
| Nmar_1250 | cytoplasmic membrane BCP | - | - |
| **Thiol-disulfide oxidoreductases** | | | |
| *N*. *maritimus* | Annotation | AR1 | AR2 |
| Nmar_0639 | DSBA oxidoreductase | 88 (AR1_654) | 86 (AR2_660) |
| Nmar_0655 | DSBA oxidoreductase | - | - |
| Nmar_0829 | DSBA oxidoreductase | 94 (AR1_911) | 83 (AR2_924) |
| Nmar_0881 | DSBA oxidoreductase | 92 (AR1_995) | 85 (AR2_1004) |
| Nmar_1140 | DSBA oxidoreductase | - | - |
| Nmar_1143 | DSBA oxidoreductase | - | - |
| Nmar_1148 | DSBA oxidoreductase | - | - |
| Nmar_1150 | DSBA oxidoreductase | - | - |
| Nmar_1181 | DSBA oxidoreductase | 94 (AR1_1347) | 92 (AR2_1347) |
| Nmar_1658 | DSBA oxidoreductase | - | - |
| Nmar_1670 | DSBA oxidoreductase | - | - |

* Locus_tag of AR1 and AR2 draft genomes in parentheses

** Genes absent in the AR1 or AR2 draft genomes
